# Patient and practitioners’ views on the most important outcomes arising from primary care consultations: a qualitative study

**DOI:** 10.1186/s12875-015-0323-9

**Published:** 2015-08-22

**Authors:** Mairead Murphy, Sandra Hollinghurst, Katrina Turner, Chris Salisbury

**Affiliations:** Centre for Academic Primary Care, School of Social and Community Medicine, University of Bristol, Canynge Hall, 39 Whatley Road, Bristol, BS8 2PS UK

## Abstract

**Background:**

Primary care clinicians often address multiple patient problems, with a range of possible outcomes. There is currently no patient-reported outcome measure (PROM) which covers this range of outcomes. Consequently, many researchers use PROMs that do not capture the full impact of primary care services. In order to identify what outcomes a PROM for primary care would need to include, we conducted interviews with patients and practitioners. This paper reports these patient and practitioners’ views on the outcomes arising from primary care consultations.

**Methods:**

Semi-structured interviews were held with 30 patients and eight clinicians across five sites in Bristol. Interviews were audio-recorded, transcribed and analysed thematically. We used a broad definition of health outcome as ‘the impacts of healthcare on health, or a patient’s ability to impact health’ to identify outcomes through this process.

**Results:**

10 outcome groups were identified. These occupied 3 domains:

Health Empowerment: These are the internal and external resources which enable patients to improve their health. This involves 1) patients’ understanding of their illnesses, 2) ability to self-care and stay healthy, 3) agreeing and adhering to a patient-clinician shared plan, 4) confidence in seeking healthcare and 5) access to support.

Health Status: This involves 6) reduction of symptoms and 7) reducing the impact of symptoms on patients’ lives.

Health Perceptions: This involves 8) patients’ satisfaction with their health, 9) health concerns, and 10) confidence in their future health.

The structure, organisation and nature of primary care means it can affect all 3 domains.

**Conclusions:**

No existing PROM captures all these outcomes. For example, many *health empowerment* PROMs do not consider patient preference on empowerment. Many *health status* tools are not responsive to changes resulting from primary care. *Health perceptions* PROMs have generally been designed for measuring personality traits rather than outcomes.

This study provides a platform for designing a new PROM containing outcomes that matter to patients and can be influenced by primary care. Such a PROM would greatly enhance the value of primary care research.

**Electronic supplementary material:**

The online version of this article (doi:10.1186/s12875-015-0323-9) contains supplementary material, which is available to authorized users.

## Background

Different models of primary care are evolving in different countries, as primary care services seek to address changing population and service needs as well as public expectations.

In recent years, general practices in the UK have experimented with new models of care to improve access, [1–3] there has been increased use of nurses to substitute for doctors, [4] and GP out-of-hours services have been contracted out. There has also been a shift in continuity of care, as patients now register with a practice rather than a named GP [5]. In the US, the patient-centred medical home has been tested through a series of pilot projects, and widely endorsed as having the potential to increase access, quality and efficiency of primary care services [6]. The 2010 Patient Protection and Affordable Care Act has further increased the focus on primary care services by promising expanded access through extending health service coverage, provider payment and service delivery reform [7]. At the same time, primary care services globally have been challenged by the increasing numbers of people living with multiple long-term conditions [8, 9], and new interventions are being developed to support this population [10–13].

These alternative configurations of primary care are evaluated through research trials designed to assess their effectiveness. Such trials routinely use patient-reported outcome measures (PROMs), i.e. questionnaires that capture outcomes as experienced by the patient completing them. An ‘outcome’ is the change in a patient's health status which is attributable to preceding healthcare [14], and PROMs provide an invaluable source of evidence for this change from the patient’s point of view [15].

Many PROMs are disease-specific, that is, tailored to the symptoms and impacts on function of a particular condition [16]. These are of no value in primary care studies where patients could have a wide-range of conditions. As a first contact, comprehensive and co-ordinating service, [17] primary care services require a *generic* PROM: one which can be administered across a population, regardless of condition. Such a PROM should be suitable for use in large-scale trials and capable of measuring outcomes over various time periods. It should be based on outcomes that matter to patients, and that are within the influence of primary care clinicians. It should also be “responsive” to change: i.e. able to detect changes that matter to patients over time [18].

The two most commonly used generic PROMs are the EQ-5D [19] and the SF-36 [20]. The EQ-5D is a utility measure [21] that contains five categorical questions focussing on physical and social function, and physical and mental health symptoms [19]. The SF-36, also measures function, and physical and mental symptoms, but does so through a more comprehensive list of questions. The SF-36 is more responsive to change than the EQ-5D [20–22]. However, both PROMs often show no change resulting from interventions in primary care [23–25]. This is, in part, because symptomatic problems are often self-limiting [26] and also because primary care patients frequently present with problems unrelated to symptoms or function [27]. In addition, most primary care patients have multiple long-term conditions [8, 9, 28] and, as their function may not improve, experts have suggested the need for a new primary care PROM that measures wider outcomes, such as a sense of control and the ability to self-care [29].

This is not a new problem. The Measure Yourself Medical Outcome Profile (MYMOP) was designed to address the apparent lack of sensitivity of generic PROMs in primary care [30]. Through allowing patients to define the symptoms and activities to be measured, MYMOP shows change when other PROMs do not [25]. However, it still focusses on symptoms and function and its individualised nature means it has to be administered at interview with a clinician, which means it is pragmatically unfeasible for use in most research trials. The Patient Enablement Measure (PEI), in contrast, encompasses broader outcomes. Based on the concept that primary care exists to ‘enable’ patients, it contains six questions including coping, understanding and confidence in health [31]. It has been well-validated for primary care, and is short and acceptable to patients and practitioners [32]. However, as well as omitting symptoms and function altogether, it is designed to measure the outcome of individual consultations. For many primary care patients, outcomes will only become apparent after multiple consultations, comprising an episode of care [33].

MYMOP and PEI were both developed nearly 20 years ago. More recently, leading commentators have observed that measurement of whole-person health outcomes in primary care has been eclipsed by an increasing prioritisation of individual disease management indicators [17, 34] and qualitative research has found that chronic conditions care is often dominated by a biomedical agenda over a patient one [35]. There is a need for a renewed focus on PROMs for primary care. Without a generic PROM, which is reliable, valid and responsive to change, it is impossible to assess the outcome of new configurations of primary care services from a patient’s perspective.

This article details findings from interviews held with patients and practitioners to explore their views on the most important outcomes arising from primary care consultations. This will be used in the future as the framework for a patient-reported outcome measure for primary care.

## Methods

### Overall design

We used a qualitative descriptive approach [36, 37], conducting semi-structured interviews to enable patients and practitioners to raise issues that were salient to them. This approach allowed the researcher to stay close to the data, directly reflecting the views of the participants. We also employed techniques from grounded theory, including simultaneous data collection and analysis and constant comparison, to ensure we reached data saturation [38]. This is described by Sandelowski as qualitative description with “grounded theory overtones” [37].

### Research setting

The research setting was the National Health Service (NHS) in the UK. We purposefully sampled and recruited five sites in Bristol in that varied in terms of consultation type: 3 GP practices, 1 walk-in centre and 1 GP out-of-hours service. We used the Index of Multiple Deprivation (IMD) for 2010 to select 3 health centres in areas of varying deprivation. The IMD is a locality-based indicator including seven domains: income, employment, health and disability, education skills and training, barriers to housing and services, living environment, and crime. These are then aggregated and weighted to calculate the index [39].

Information by site is shown in a tabular format in Table 1.

**Table 1:** Description of 5 participating sites

	Site 1	Site 2	Site 3	Site 4	Site 5
Service Model	Health Centre	Health Centre	Health Centre	Walk-in Centre	Tele-health and out-of-hours provider
Index of Deprivation	Lower quartile	Median	Upper Quartile	Lower quartile	n/a
Staffing	9 doctors, 6 nurses and 4 other clinical staff.	8 doctors, 6 nurses and 4 other clinical staff.	9 doctors, 5 nurses and 4 other clinical staff.	5 doctors, 9 nurses and 2 other clinical staff.	Not known
Recruitment Dates	Fri 28^th^ Jun 2013 Wed 17^th^ Jul 2013	Thu 1^st^ Aug 2013	Mon 8^th^ Jul 2013	Mon 22^nd^ Jul 2013	
Interview Period	10^th^ Jul – 16^th^ Sep 2013	28^th^ Aug – 10^th^ Sep 2013	29^th^ Jul – 30^th^ Aug 2013	17^th^ Jul – 31^st^ Jul 2013	21^st^ Oct – 31^st^ Oct 2013

### Data collection

We interviewed both patients and clinicians, as we wanted to identify patient-relevant outcomes which clinicians felt able to influence. Two topic guides, one for patients and one for clinicians, were used to ensure consistency across the interviews. To inform these topic guides, we employed a working definition of a health outcome as follows:

*Primary Care Health Outcome*: any effect of primary care on a patient’s health status or ability to impact health status which persists outside the consultation.

The World Health Organisation definition of health is implicit in this: health as “a state of complete physical, mental and social well-being and not merely the absence of disease or infirmity” [40]. We widened the definition of health outcome to include ability to impact health status as well as health status itself. We also defined outcome as effects which persisted beyond the consultation to distinguish it from *experience*, which we defined as the patient’s perceptions of the consultation itself.

#### Patient interviews

Patients were approached in waiting rooms by the researcher [MM] in the 3 general practices and the walk-in centre, and recruited by letter from the GP out-of-hours service provider. Patients who were willing to take part supplied contact details and information about their age, ethnicity, sex and long-term-conditions. This information was used to purposefully sample patients to ensure maximum variation in relation to the last four variables.

The interviews were conducted face-to-face in the patients’ own homes or other location of their choice, or by telephone, with written consent taken immediately prior to the interview. The topic guide was designed to elicit what outcomes patients sought from primary care, and was extended during the course of the interviews to incorporate themes identified in earlier data collection. If patients tended to talk about “helpful” or “unhelpful” consultations in terms of their experience of care, rather than the outcome, the interviewer used probing questions such as, “did you do anything differently as a result of that experience?” or “did that affect your health or well-being?” The final topic guide is attached in Additional file 1.

#### Clinician interviews

Clinicians were recruited from the same sites via their respective practice managers. A maximum variation sample was sought across gender and years of experience. Clinicians were interviewed face-to-face in their respective health centres or by telephone, and gave consent in writing. The topic guide was designed to elicit what outcomes they thought their patients needed and wanted, and they could influence. In a similar way to the patient interviews, the topic guide for the practitioners changed over the course of the interviews in order to incorporate emerging themes. The final clinician topic guide is attached in Additional file 2.

### Analysis

Patients and practitioners were interviewed until data saturation was reached, i.e. when no new themes arose from new data [38, 41]. The interviews were audio-recorded and transcribed verbatim. One researcher [MM] read and re-read the interview transcripts from both patients and practitioners, in order to gain an overall view of the accounts given, to identify themes and develop an initial coding frame. As the purpose of the study was to inform a PROM, we structured the coding frame so that it included the heading of ‘outcomes’, under which all data relating to patient and practitioners’ views of outcomes were coded. Similar outcome themes emerged from both patient and practitioner interviews, so we decided to have a single coding frame, which could be applied to both sets of interviews. MM developed the initial coding frame using the qualitative data analysis package NVivo10™. The codes evolved as each transcript was read, with some codes being subsumed by others, some being disaggregated and some being renamed as the researcher’s understanding of the data evolved [38].

To finalise the coding frame, two co-investigators [CS&SH] independently reviewed 4 interview transcripts and identified emerging outcome themes. CS, SH and MM then discussed these themes and agreed on the coding frame. MM then electronically coded all the interviews, and re-coded those she had coded earlier, according to this coding frame. Constant comparison was used, whereby codes and themes emerging in early interviews were compared against themes emerging in later interviews. NVivo queries were used to identify if there were any outcomes which were raised only by clinicians, only by patients or types of patient (e.g. older patients with long-term conditions).

### Ethical considerations

The study received ethical approval from Nottingham 1 research ethics committee, in particular for issues of informed consent, data confidentiality and protection. It was supported by the NIHR Clinical Research Network (CRN) and included on the CRN Study Portfolio [42].

## Results

### Participants

Thirty patients and eight clinicians were recruited across the five sites. In one of the participating health centres clinicians did not respond to the recruitment call. Clinicians from two General Practices over and above the original five sites, from the median and upper quartile index of multiple deprivation were included to increase the number of interviewees. Patient and clinician demographic information is shown in Tables 2 and 3 respectively.

**Table 2:** Characteristics of patient interviewees

Characteristic	Number Interviewed
Gender	
Female	16
Male	14
Age Bracket	
18–34	5
35–54	10
55–64	4
65–74	5
75+	6
Ethnicity	
Asian	2
Black	3
mixed race	1
White	24
Number LTCs	
No long-term conditions	5
One long-term condition	13
> One long-term condition	12

**Table 3:** Characteristics of clinician interviewees

Characteristic	Number Interviewed
Gender	
Female	6
Male	2
Profession	
Doctor	6
Nurse	2
Year qualified	
1981–1990	4
1991–2000	2
After 2000	2

### Overview of outcomes

We identified 31 outcomes during the analysis of the patient and practitioner interviews. The full list of these outcomes can be found in Additional file 4. The outcomes were categorised into three hypothesised domains: Health Empowerment, Health Status and Health Perceptions. In total, there were 10 outcome groups within these domains. These are shown in Table 4.

**Table 4:** Domains and outcomes of primary care

Domain	Outcome Group
Health Empowerment	1: Understanding of illness, conditions or problems
	2: Ability to self-care and stay healthy
	3: Confidence in Seeking Healthcare
	4: Access to Support
	5: Following a patient-clinician shared plan
Health Status	6: Symptoms
	7: Effect of symptoms on life
Health Perceptions	8: Satisfaction with health
	9: Health Concerns
	10: On track for the future

We defined Health Empowerment as resources patients have access to both internally and from their environment that enable them to positively impact their health. The analysis suggested that as comprehensive, coordinating first point of access, Primary Care has a strong influence on this.

Health Status includes the more commonly measured outcomes of symptoms (physical and emotional), side-effects, and the impact of symptoms on life. Although these are important reasons for providing healthcare, they are not outcomes that primary care can always influence.

Health Perceptions is how patients feel about their current and future health. While this clearly depends on health status, our research suggests that clinicians can also influence this by increasing patient understanding and explaining the scope and limitations of care.

### Description of outcomes

#### Health empowerment outcomes

##### Understanding of illness conditions or problems

*“I didn’t feel I was getting all the answers.” (Patient 17)*

Both patients and clinicians described increased patient understanding of illnesses or ‘problems’ as a positive outcome from primary care. This was often expressed by patients in terms of new or changing symptoms using terms like “understanding”, “knowing what is going on” and “getting answers”. Clinicians expressed this outcome mainly in terms of “diagnosis” and “prognosis”. Clinicians believed that increased understanding could help patients to research their condition, or simply feel reassured that serious illness was not indicated. Two clinicians felt the “naming of the unknown” was an end in itself for many patients.

Exceptions to this included two elderly patients with long-term conditions, who both expressly stated no desire to increase understanding of their condition. When asked why, one 86-year-old responded:

*“I don’t worry about nothing …You know, I just take it, if I’m gonna die, I’m gonna die and that’s the way I look at it.” (Pt21)*

However, this patient did acknowledge that he required a certain level of understanding about his long-term conditions, including what each of his medications was for and how he needed to take them.

##### Ability to self-care and stay healthy

*“If I get a recurrence I know exactly what to do” (Patient 12)*

This outcome group captures patients’ ability to improve and maintain their own health; and includes firstly the ability to manage symptoms and secondly the adoption of healthy behaviours. Just over one third of patients interviewed described symptom self-management as a recent positive outcome. A woman in her 60s who had consulted with a rash explained this in terms of information:

*“I think that it is a kind of bi-product of going to see your doctor that you pick up information and that helps when something similar arises again.” (Pt6)*

One man described this outcome in terms of having medication available. With regards to his doctor pre-prescribing for gout he said:

*“I've got a nice, erm, stock of medication now … so if I get a recurrence I can, I've got it on hand immediately, to use.” (Pt12)*

The same man also described the second related outcome as knowing what lifestyle he should adopt to prevent future attacks:

*“Basically he [the GP] said that I should cut down drinking and more importantly […] there are lots of dietary things that I know I should avoid, which I wasn’t aware of before.” (Pt12)*

When asked if this information was helpful, this patient responded “Oh yes, yes”. However other patients described receiving lifestyle advice as *unhelpful*. One man described consulting with pain from a previously diagnosed ulcer. The GP advised him to *“go away, stop smoking and then come back and see me” (Pt14)*

*Pt14:I slammed the door and I went.*

*INT:Did you go away and stop smoking?*

*Pt14:No I went bleep, bleep, bleep, bleep as I walked out the clinic and I never went back there again*.

Despite receiving information on how to stay healthy, the patient did not change his behaviour, or even increase his knowledge as an interim step. One GP also commented healthy behaviour as something that GPs attempted to influence, sometimes with little success.

*“The incentivisation is very big on GPs doing brief interventions […] the research says it’s effective… [but] I never get that much feedback from the patients when I mention this; I never get this sense of enlightenment or awareness.” (GP3)*

This underlines the importance of measuring outcome (actually increasing the patient’s knowledge) rather than process (simply passing over the information).

##### Confidence in seeking healthcare

*“I just want to keep him engaged with the service” (GP3)*

This outcome encapsulates patients’ trust in clinicians and engagement with health services. It involves a range of sub-outcomes incorporating confidence in accessing care, and trust that clinicians will listen to them and help them.

An example arose when a GP described a male patient attending with violent anger issues and marijuana use. The GP did not press him after he declined a referral to the community drugs service, explaining:

*“He didn’t come for help with giving up the marijuana; he came because he was afraid he was going to hurt himself, or hurt someone else […] and so, if you don’t address that point, he’ll never come back and see you, and the whole time has been wasted.” (GP3)*

This GP believed that the priority was addressing the patient’s immediate problem, and ensuring that the patient was confident in returning to see the doctor. Patients seemed to only notice this outcome in its absence, and therefore tended to describe it negatively. One patient, who had a series of chronic problems, which she believed were not being taken seriously by GPs, said:

*“For a long long time I just didn’t go because I was worried that I’d be packed away […] it wasn’t until things started getting really bad [that I attended]” (Pt15)*

This patient had described how seeing a series of GPs who did not address her chronic problems led to her feeling alienated. She later described how establishing a relationship with a particular GP increased her confidence, as mutual trust was built up over a period.

##### Access to support

“That was a really amazing service that I didn’t even know existed” *(Patient 4)*

This outcome captures the access to support facilitated by primary care, and includes support that meets psychological and social needs as well as practical needs. Eight patients raised this as a desired outcome.

A GP explained how the role of primary care was greater than informing patients about available support, and included a process of helping patients understand the support they need.

*“I think they [patients] initially want to sort out what it is and then [what] you can offer them […] Domestic violence is a good example, people often don’t recognise the issue that they have […] we can support them with referrals to Next Link who will just pick that up and deal with it in a really great way.” (GP6)*

According to this GP, sometimes patients do not understand their own issues sufficiently to research and access support themselves. The role of the GP is therefore more than managing a referral gateway, but includes working with patients to understand the root of their problems, before signposting or facilitating access to a service that can help.

##### Following a patient-clinician shared plan

*“I need some advice about … the plan of action.” (Patient 15)*

Both patients and clinicians described an agreed plan of action that the patient could adhere to as an important outcome. For some patients, collaboration was an import element in this. In other patients this was less important. These tended to be older patients who viewed the GP as an expert whose advice should be followed. This group of patients still wanted an agreed plan, although they required less input in this plan provided they trusted their GP.

#### Health status outcomes

##### Symptoms and side effects

*“The outcome I want is the pain to go away” (Patient 26)*

Four sub-outcome themes emerged under this heading, including pain or discomfort, depression or anxiety, side-effects of medication and other symptoms.

Pain relief was one of the most important outcomes desired. One patient described how, for him, this outcome ultimately overrode the other interim outcomes he also desired. He had experienced sudden pain in his shoulder, and had dialled 111. The pain was diagnosed as a trapped nerve.

*“The outcome I want is the pain to go away but. […] … nobody’s suggested anything to cure the problem […] They said go back to your GP and she’ll monitor, and I thought, well, yeah … thank you very much.” [sarcastically] (Pt26)*

The patient had described how initially, the possible illnesses indicated by a sudden onset of pain was of greater concern to him than the pain itself. After diagnosis, his concern abated, but the pain remained. He felt that his clinician had lost sight of the importance of pain relief as an outcome, and seemed to feel their job was complete at diagnosis. About one third of patients expressed this as an outcome they had recently wanted.

Nearly half of the patients referred to reducing the side-effects of their medication as a desired outcome. Many patients described working with their clinicians to arrive at the best combination of medication which balanced symptoms with side-effects.

Some patients described their primary care clinicians as helping them to relieve anxiety, depression or feeling “down” or “low”. Patients described being helped in 3 main ways; by counselling from their GP or nurse, referral to specialist support, and medication. The majority of the patients said their primary care clinicians helped by “listening” (Pt18), “talking to [them]”(Pt24) or “having a chat”(Pt21), which in turn helped the patients to “set their mind at ease” (Pt21), “calm down”(Pt29) or “feel positive”(Pt24). One patient described this, when asked to say more about how his asthma nurse “helped” his mood:

*“She makes you feel positive just by talking to you I suppose. Sometimes you break down and you can’t cope … So I’ve got ways of either getting help, I can talk to my partner but I don’t want to stress her out because she’s got a full-time job … I can talk to my friend, Dave, or I can talk to her [asthma nurse] .. She just helps me by talking to me you know”.* (Pt24)

The patient describes how the outcomes of “feeling positive” is helped by the ongoing relationship he has with this nurse, and by the relatively ready access he has to primary care services.

##### Effect of symptoms on life

*“I’m not sure whether I can continue like this” (Patient 15)*

Increased ability to manage in daily life was an outcome desired by a number of patients. Three sub-outcomes emerged: patient’s ability to carry out normal activities, patient’s ability to enjoy life, and the effect of illness on other people in life. This theme is wide-ranging, in that these outcomes can be influenced by many different conditions or problems, and by a large number of different actions from the clinician. It also encompasses physical, emotional and social function. One patient, who was waiting for surgery for a rectal prolapse, spoke with frustration about how she could not live normally because of her symptoms.

*“I’ve got no control of urine um … faeces, anything. It can all just decide to happen […] I can’t carry on … I can’t, I need to go abroad and visit my daughter” (Pt16)*

This woman wanted correction of the prolapse through surgery and thereby focussed on mitigating effects through curing illnesses. Some other patients and clinicians, in contrast, referred to developing coping mechanisms or “Giving patients strategies” (GP2) to improve function, rather than providing a cure, particularly in the context of chronic illness.

A number of patients with mental health problems described themselves as unable to manage in daily life. This feeling was recognised by practitioners:

*“often people with depression they don’t know what they want, they just know they can’t go on, well, to quote: “I can’t go on like this, doctor”” (GP6)*

This doctor then described his role as working with the patient to understand the nature of the problem, and agreeing a plan to help improve the situation.

#### Health perceptions outcomes

Through the interviews, patients described their perceptions of their health. Their accounts indicated that, although satisfaction with health depends on the actual health state as well as expectations and values, it can also be influenced by primary care directly.

##### Perception of current health

*“I am now 76, so can expect things to start to wear out.” (Patient 26)*

Some patients felt positive about their health, despite having some health problems. These patients generally believed they received good primary healthcare, and had realistic expectations grounded in an understanding of their conditions.

One elderly man described his current health state:

*“I’ve got no long term problems, well except I’ve had a problem with my vertebrae in my spine for a long time […] There’s nothing they can do about it I believe, I did even go and see a consultant privately about this and they came out with the same answer […] I got initially from GP … it’s just wear and tear.” (Pt26)*

This elderly man was happy with his health for his age, despite his health problems. Previously in the interview, he described the care he received from both his GP and his practice nurse as excellent, and explained that he had confidence in his GP as she picked up his wife’s cancer at an early stage. The consistent diagnosis between healthcare professionals had added to this confidence, implying that primary care did have some influence in this case.

Many patients who were dissatisfied with their health believed that it could be improved through intervention. One patient who felt she needed an operation said “my life is hold” (P16). This patient’s perception of her health was influenced by an internal benchmark of how she felt her health could be if she was operated on. She expressed frustration with what she perceived as poor communication from her GP and poor co-ordination of health services.

#### Health concerns

“*She [the nurse] left me very reassured.” (Patient 13)*

Nearly half of patients described alleviating concern about serious illness as a desired outcome of a recent primary care consultation. Some patients described themselves as only mildly worried, using phrases such as “I just wanted to make sure”(Pt1) or “I just wanted reassurance” (Pt14), but most of the patients described themselves as moderately or extremely concerned on at least one occasion in the recent past. Four were concerned that symptoms might indicate cancer, four that they might indicate a heart attack, and four other serious illnesses.

#### On track for the future

*“I feel that he [my doctor] has set me on the path” (Patient 15)*

This outcome group includes patients’ belief that they are on the right path to dealing with their problems and that they are dealing with the root of their problems. An essential element of primary care is early management of health problems: ensuring patients get the right diagnosis and treatment to reduce the risk of future problems [43]. Such treatments do not always make patients feel immediately better, and indeed may make them feel worse in the short term. As one GP put it:

*“If you get admitted to hospital, you’re being admitted for a specific treatment for a thing, and you are actually expected to be healthier at the end of that particular episode … which is not the case in general practice [..] for many conditions, your role might be to determine what it is, and set them on the right course. But, actually, chances are they’re going to get worse before they get better. (GP3)*

An important element of patients feeling like they are on the right ‘path’ is their sense that the root of their problems is being addressed. Perhaps because they only noticed this outcome in its absence, patients often described this negatively. For example, one patient, who has a complex genetic condition causing him to feel depressed sometimes, described this as follows:

*They prescribe you anti-depressants. Anti-depressants are not the answer because […], that doesn’t help you sort things out […] been offered them, never taken them. I’ve seen people that have taken them and that just masks the issues. (Pt24)*

This patient felt the doctors focussed on his immediate symptoms without addressing the root of the problem. He felt that the solution provided by GPs was “masking” his issues, and that if he could try to deal with those issues, this would be more effective in dealing with his depression.

GPs also thought that addressing the root of patients’ problems was important. However, many GPs thought that patients were reluctant to address the root of problems. One GP described this in terms of balancing the conflict between the patient’s immediate and underlying problems:

*If they’re your patient, there’s continuity, then you can pin them down, you can spend five minutes going, look, I realise you want to talk about your anxiety again, but we’ve done that. We need to talk about the root of this; let’s talk about your marijuana addiction. (GP3)*

This GP emphasised the need for continuity in complex patients. He described how, in initial consultations he will address the problems patients ostensibly present with, and only after developing a rapport with a patient (a word he used later in the interview), will he introduce the deeper issues that may be underlying cause of the patients problems.

## Discussion

### Key findings

This study describes the outcomes arising from primary care, as identified by patients and practitioners. Ten outcome groups occupied three separate domains: health empowerment; health status; and health perceptions.

Although many of the outcomes that emerged inductively in this model have been discussed elsewhere, and are captured in well-validated patient-reported questionnaires, [22, 44, 45] this study differs from other models of outcome in three key ways. Firstly, by asking patient what outcomes they sought from clinicians, and clinicians what they sought for patients, it explicitly sought to identify outcomes that can be influenced by primary care. It thus excludes some outcomes that may be important to patients, but that they do not believe primary care substantially impacts (overall quality of life was one such domain). Secondly, it has used a broad definition of health outcome, as improvement in health status or ability to impact health status. It thus includes outcomes which are missing in other questionnaires. Thirdly, it has only included outcomes which patients can directly perceive, and therefore are suitable for inclusion in a PROM.

A description follows of how each of the 3 domains is relevant to a PROM for primary care, and the strengths and limitations of existing PROMs covering these domains are discussed.

### Health empowerment

The Health Empowerment outcomes describe the resources patients have access to, both within themselves (internally) and within their environment (externally) to positively impact on their health.

The term empowerment is already used in healthcare literature. Although it does not have a consistent definition, it generally has a greater focus on the internal than the external, combining elements of self-efficacy and control [46]. McAllister et al. described the empowered patient as one who has “some control” over her health, as opposed to being a “passive recipient” of healthcare. They argued that empowerment is a valuable outcome for patients with long term conditions, even if it does not confer any health benefits [46].

This view is also implicit in many patient-reported empowerment questionnaires. For example, Hibbard’s 13-item Patient Activation Measure is a uni-dimensional Guttman scale [47] which assesses patient “self-reported knowledge, skill, and confidence for self-management of one’s health” [48]. Consider the item, “I know the different medical treatment options available for my health condition”. Increasingly positive responses to this question mean more empowered (or activated) patients, regardless of patient preference.

Such a prescriptive approach to empowerment was not fully supported by this study. Many patients did not want or need to be as ‘empowered’ as others. This is consistent with previous research showing that certain patients purposely limit their health knowledge as a strategy for feeling in control [49]. However, when directly asked, all patients in this study did acknowledge the need for a certain level of understanding, knowledge and ability to self-care. This underlines the need for a new patient-reported tool in this area, which measures empowerment more flexibly, recognising the outcomes as positive, but varying in importance depending on the person.

We found that some clinicians prioritised maintaining patient’s confidence in seeking healthcare over outcomes of improved self-care and healthy behaviour. Hunter et al., similarly found that many clinicians avoid conversations about behavioural change because of fears they might “lose” the patient through harming the patient-clinician relationship [50]. Because we explicitly included *both* confidence in seeking healthcare and ability to self-care/stay healthy as empowerment outcomes, any PROM developed from our study will test the extent to which either is influenced by primary care.

### Health status

The health status outcomes include symptoms, and the effect of symptoms on life. The 1986 WHO Ottawa charter described health as “a resource for everyday life, not the objective of living”: i.e. the effects of symptoms on patients’ lives are more important than the symptoms themselves [51]. In line with this, although most generic PROMs include ‘Pain’ and ‘Anxiety / Depression’ as outcomes in their own right, outcomes measurement has moved beyond measurement of symptoms with a greater focus on the effects these symptoms have [22, 44, 45].

In this study, many patients expressed their desire for improved physical, emotional and social well-being in terms of reducing the effects of symptoms. The SF-36 captures function in a similar way to this. For example, in the SF-36 social function is captured in questions such as: “to what extent has your physical health or emotional problems interfered with your normal social activities with family, friends, neighbors, or groups?” [20]. Given this focus on the impacts of physical and emotional health, it is not surprising that the SF-36 is the most responsive to change of the leading generic measures of symptoms and function [22]. Tapping into the specific sub-domains and the language patients used in the interviews will allow for development of a PROM which is maximally responsive to these outcomes as they arise in primary care.

Improvement in symptoms and function may be seen as the ultimate goal of health-care. However, timing is important in this and experts have pointed out that, in primary care, focussing solely on symptoms and function may create incentives for healthcare providers to focus on short-term gains at the expense of longer-term outcome [33]. This is because interventions to improve long-term health (for example, statins) may make patients report worsened symptoms and function in the short term. In the context of primary care, there is therefore a need to measure patients’ beliefs about their health and future health. This is covered by the overall domain of Health Perceptions.

### Health perceptions

Patients in this study described themselves as feeling satisfied or unsatisfied with their current state of health. This concept, which emerged inductively from the data, has resonance with the concept of “health perceptions” described in Wilson and Cleary’s model of health-related-quality-of-life [52] and with ‘satisfaction with outcome’ in Starfield’s model of primary care outcomes [53].

This construct is often measured through a single-item self-rating of health. Proponents of measuring health perceptions as separate from symptoms and function argue that it is a subjective concept which has as much to do with a person’s feelings and beliefs as their actual health status [54].

Questionnaires designed to capture this concept in a multi-item format have had limited use as outcome measures. For example the RAND health perceptions questionnaire [22] which contains 33 questions in 6 sub-scales, has shown high stability over time, suggesting that it may be more useful as a trait indicator than an outcome measure that is responsive to change [22]. There is, however, some evidence that health perceptions can be influenced by interventional changes in sicker populations [55] and the investigators suggest that the role of medical interventions in shifting illness perceptions is an important and under-researched area [56]. By identifying the particular sub-domains of health perceptions that patients and clinicians described as outcomes of primary care, this study provides a platform for designing a PROM which will test the hypothesis of whether these outcomes can be influenced by interventions in primary care.

### Strengths and limitations

The model described has a number of strengths for taking forwards to a generic PROM. The definition of outcome used has allowed inclusion of both interim and final outcomes, thereby balancing sensitivity to change with impact on patients. The model is grounded in data from a maximum variation sample of patients and clinicians.

One limitation of this sample is, as with any interview-based research, it was limited to people who agreed to an unpaid interview, thereby forming a self-selecting group who either displayed a level of altruism, or had a particular ‘story’ about primary care which they wanted to tell. Although a maximum variation of sites was used, these were limited to Bristol only. Patients over 80 proved difficult to recruit (there was only 1 participant in this group) and a higher number of over 75 s were sought as a result of this. The sample was also 80 % white. While representative of the UK population, this may limit the study’s findings for countries with a different ethnic composition. Finally, the sample of clinicians included a higher number of doctors than nurses, and women than men. However, as we continued sampling until data saturation was reached, it is not thought that these issues affected the results unduly. The maximum variation sample was representative of general practice for the number of patients who had long-term conditions [9] and by age-bracket [57] apart from over 80 year-olds. Because some ethnic minorities are higher users of primary healthcare [58], are less satisfied with healthcare [59, 60] and have greater health inequalities [58, 61] the researcher purposively sampled a higher number of this population.

A second limitation is based on the premise of deriving a single set of outcomes from a diverse group of people: as with any generic set of outcomes, they vary in relevance and importance between individuals. We believe that this is an unavoidable challenge. Although establishing mechanisms to address relative importance and redundancy within patient questionnaires are not easy, the alternative is to bypass the problem by failing to measure generic outcome in primary care and therefore to be unable to assess the benefits for patients of alternative models of provision of primary care.

## Conclusions

This study describes patients’ and clinicians’ perceptions of the outcomes arising from primary care, which can be used as a basis for a PROM.

There are generic PROMs in existence which cover aspects of these constructs. However, no existing PROM covers the all the outcomes described above. Even the PROMs which provide partial coverage of these constructs do not do so in a way which would properly measure outcome from primary care. For example, many *health empowerment* PROMs do not build in the patients’ desire for empowerment. Most *health status* tools ignore side-effects, and many are not responsive to change in primary care. *Health perceptions* PROMs have generally been designed as trait measurement tools rather than as outcomes tools, and therefore do not focus on outcomes which are responsive to interventional change. Using a combination of these existing health measures to assess outcome in primary care is, therefore, not only long and burdensome for the patient, but also *excludes* important aspects of the relevant constructs, and *includes* aspects which are likely to be unchanged by primary care.

By identifying the particular sub-domains that both patients and clinicians described as outcomes of primary care, this study provides a platform for designing a PROM which will test the hypothesis of whether these outcomes can be influenced by interventions in primary care.

## Additional files

Additional file 1:
**Patient Semi-Structured Interview.** (DOCX 14 kb) Additional file 2:
**Clinician Topic Guide.** (DOCX 12 kb) Additional file 3:
**Patients recruited per site.** (DOCX 12 kb) Additional file 4:
**List of Identified Outcomes.** (DOCX 16 kb)

## Abbreviations

EQ-5D: EuroQol 5D
MYMOP: Measure yourself medical outcomes profile
NIHR: National institute of health research
NIHR CRN: NIHR clinical research network
NIHR SPCR: NIHR School for primary care research
PEI: Patient enablement instrument
PROM: Patient reported outcome measure
SF-36: Medical outcomes study short form 36
WHO: World health organisation
WHOQoL-BREF: World Health organisation quality of life – brief questionnaire
