# Supplementary material for: Patient and practitioners’ views on the most important outcomes arising from primary care consultations: a qualitative study
Source: BMC Fam Pract. 2015 Aug 22;16:108. doi: 10.1186/s12875-015-0323-9 (PMC4546201; doi:10.1186/s12875-015-0323-9)
Supplement: Additional file 1: — Patient Semi-Structured Interview. (DOCX 14 kb) [file 12875_2015_323_MOESM1_ESM.docx]

***Patient Semi-Structured Interview:***

***Topic 1: Last consultation – outcomes desired / achieved***

Can you tell me about your last appointment, why you went and whether it achieved what you wanted?

**Probes:**

How were you / have you been affected by that problem?

How long had you been feeling like that before you contacted them?

What did the doctor do?

Did the consultation achieve what you hoped?

It sounded like you found the consultation helpful **OR** Did it help? How?

***Topic 2: Long Term Conditions and Goals:***

You indicated you do suffer from a long-term condition – how does that affect you?

What are your goals for your LTC/health?

How does the healthcare you get help with that?

How much do you see as something you have to manage yourself, and how much does your doctor help?

***Topic 3: Previous Consultations – Good and bad outcomes***

Can you describe any previous consultations that have been particularly helpful? Why was it so helpful?

What about a consultation that was really very unhelpful? Can you think of any like that?

***Topic 4: What does good care look like – how does it affect outcome***

Do you prefer to see the same doctor? Why? How does that affect outcome?

Do you find it easy to access primary Care? How does that affect outcome?

What do you think good care from your GP / nurse looks like? How does that affect outcome?

What would you like to maintain / improve? (if focus on experience/process, prompt for outcome.)

Does your doctor help you get any other forms of care? How does that affect outcome?

Does your doctor provide you with any other support? How does that affect outcome?

***Topic 5: Confirmation of other themes arising***

Some other people I interviewed mentioned that their GP helped them with X.

I wonder if you felt the same – I am thinking especially about the experience Y you described.

e.g. Information, Reassurance , Referral / Medication, Symptoms, Function

***Probes for when patients talk about experience, not outcome:***

When patients talk about experience ....

- How did that experience affect you?
- Did it affect your health or well-being? (Note – it might have affected it indirectly)
- Did it take longer / shorter to recover because of that?
- Did that experience make you feel ….
  - Better in yourself / Upset or annoyed? – please say more about that
  - Worried / Reassured? – please say more about that
  - less likely / more likely to follow the doctors advice? – please say more about that
- Has it had any long-term impact on your health?
