# Supplementary material for: Patient and practitioners’ views on the most important outcomes arising from primary care consultations: a qualitative study
Source: BMC Fam Pract. 2015 Aug 22;16:108. doi: 10.1186/s12875-015-0323-9 (PMC4546201; doi:10.1186/s12875-015-0323-9)
Supplement: Additional file 2: — Clinician Topic Guide. (DOCX 12 kb) [file 12875_2015_323_MOESM2_ESM.docx]

**Clinician Topic Guide**

***Topic 1: The nature of the problem***

Describe problems with EQ-5D on GPPS, then ask – do you have a view as to what kind of domains might go into a PC PROM, apart from symptoms and function?

Follow up questions: To what extent can you really influence that outcome for your patients?

Can you think of an example when you achieved that for a patient?

Does that affect their health in the long-term?

***Topic 2: What patients value***

What do your patients seem to value in their consultations with you?

Do you think that affects their outcome? Can you think of an example?

X% rate this surgery according to the GPPS. What is the surgery doing that is providing value?

Do you think that affects their outcome?

***Topic 3: Unique practice elements***

I see you have an X based at practice (osteopath / Minor illness clinic etc.) Can you say some more about that?

How does it help patients / do patients value that?

Does it affect their outcome?

***Topic 4: Patients with long-term conditions***

I’m interested in talking about the needs of patients with long-term conditions. X% of the patients on the books of this surgery have long term conditions which give them problems in daily life.

What do you think they value most?

How do you think primary care helps them? ... point towards outcome

Can you think of any examples?

***Topic 5: 4 Cs***

Some other patients and clinicians have mentioned continuity of care. What does this mean to you? Do your patients value it? How does it help patient outcome?

Some other patients and clinicians have mentioned co-ordination. What does this mean to you? Do your patients value it? How does it help patient outcome?

What does patient-centred care mean to you? How does it help patient outcome? Do your patients value it?

***Topic 5: Themes which arose previously (patients and doctors)***

Some other clinicians I have interviewed said that they thought an important outcome they achieved for their patients was X? What do you think about that?

Is it a short-term gain, or a long-term gain?

Some patients I have interviewed …..

Go through 1 by 1

***Topic 6: Patient understanding and expectations***

Do you think your patients have reasonable expectations of what primary care can bring them?

Probe: Have you ever had an experience where you think a patient leaves your surgery unhappy with the outcome, but you think the outcome was actually the best they could have hoped for in the circumstances?

How do you deal with that?

***Topic 7: Anything Else:***

Is there anything else particularly relevant to identifying important outcomes for patients that we haven’t covered that you would like to add?
