# Supplementary material for: Patient and practitioners’ views on the most important outcomes arising from primary care consultations: a qualitative study
Source: BMC Fam Pract. 2015 Aug 22;16:108. doi: 10.1186/s12875-015-0323-9 (PMC4546201; doi:10.1186/s12875-015-0323-9)
Supplement: Additional file 3: — Patients recruited per site. (DOCX 12 kb) [file 12875_2015_323_MOESM3_ESM.docx]

Additional File 3: Patients recruited per site

|  | **IMD %ile for 2010** | **Total Patients Approached** | **Total Providing Details** | **Total Interviewed** | **Reason for difference** |
| --- | --- | --- | --- | --- | --- |
| **Site 1**  **(LQ IMD)** | 22 | 69 | 16 (23%) | 6 (9%) | 7 later declined or did not respond, 3 purposively not pursued |
| **Site 2**  **(Median IMD)** | 50 | 35 | 14 (40%) | 7 (20%) | 2 later declined or did not respond, 5 purposively not pursued |
| **Site 3**  **(UQ IMD)** | 79 | 14 | 7 (50%) | 5 (36%) | 2 later declined or did not respond |
| **Site 4 (Walk-in Centre, LQ IMD)** | 12 | 34 | 14 (41%) | 4 (12%) | 8 later declined or did not respond, 1 purposively not pursued |
| **Site 5 Telehealth OOH** |  | 77 | 15 (19%) | 8 (10%) | 7 later declined or did not respond |
