# Supplementary material for: Patient and practitioners’ views on the most important outcomes arising from primary care consultations: a qualitative study
Source: BMC Fam Pract. 2015 Aug 22;16:108. doi: 10.1186/s12875-015-0323-9 (PMC4546201; doi:10.1186/s12875-015-0323-9)
Supplement: Additional file 4: — List of Identified Outcomes. (DOCX 16 kb) [file 12875_2015_323_MOESM4_ESM.docx]

**Additional File 4: List of Identified Outcomes**

The list of themes and sub-themes below shows outcomes that patients seek from primary care and that clinicians believe that primary care can influence. As with any outcomes, the influence of primary care is a partial one, and primary care will not be able to influence all outcomes in all cases.

|  | **Outcome** | **Sub-theme** | **How Primary Care can influence** |
| --- | --- | --- | --- |
| **Health Empowerment** | 1: Understanding illness, conditions or problems | Patient understanding of illness, conditions or problems | By giving an accurate diagnosis, clear explanations, arranging diagnostic tests or specialist support as necessary and providing written information or signposting where to find such information. |
|  |  | Patient understanding likely progression of condition or illness |  |
|  |  | Patient ability to explain health problems to others where necessary | Through increasing the patient’s understanding (see above) and thereby their ability to communicate to others, or by directly communicating on the patient’s behalf with interested parties (e.g. work, school, benefits office, jobcentre, family). |
|  | 2: Ability to self-care and stay healthy | Patient ability to take action to manage symptoms | By giving clear explanations on how to manage symptoms, so that patients are confident they can self-manage in future where appropriate. By prescribing medication and making it easy for patients to access repeat medication if required. |
|  |  | Patient knowledge on how to stay healthy | Through communicating information about what lifestyle actions patients need to take in order to improve or maintain health. By doing this in a way that is individualised to each patient’s needs and ability to process and act on the information. |
|  |  | Patient’s commitment to taking responsibility for their own health | By encouraging patients to take responsibility for their own healthcare as appropriate. By doing this in a way which is responsive to the needs of each individual patient. |
|  | 3: Have a plan and adhering to it | Patient’s shared understanding with clinician on plan of action | By ensuring that they attempt to address the problems that patients present with through agreeing a plan of action which the patient understands, agrees with and has contributed to as much as he or she would like. |
|  |  | Patient’s adherence to advised or agreed plan | By ensuring that the plan agreed with the patient is one that the patient has confidence in, has the ability to follow, and has clearly understood. |
|  |  | Patient’s understanding of the rationale behind clinician’s advice | By taking time to explain to patients why they have recommended or not recommended a particular course of action, and clearly responding to questions on this so that patient understand why they have been given particular advice. |
|  | 4: Confidence in Seeking Healthcare | Patient confidence that they can access healthcare support when really needed | By ensuring patients can access primary healthcare services: i.e. easily contact the practice by telephone, and by developing systems to manage patient urgent health needs with limited capacity. By facilitating patient access to other services, including secondary care when needed. |
|  |  | Patient confidence that clinicians will do their best to help | By listening to, and believing patients, without pre-judging based on assumption or prior knowledge. By taking patient problems seriously, and continuing to try new approaches for persisting problems. By taking responsibility for patients under their care and being willing to take decision and action on the patient’s behalf. |
|  |  | Patient confidence that clinicians will listen, not prejudge |  |
|  |  | Patient confidence in the health system | By correctly using their information and communication systems, and by diligently checking referrals and results and ensuring clear lines of communication to patients. |
|  | 5: Access to Support | Patient awareness of the options available for support | Through listening to patients to understand their support needs, and informing patients about the support available. |
|  |  | Patient access to support which meets psychological and social needs | Through listening to patients to understand the nature of their problems, advising them and referring them to the support agencies available. |
|  |  | Patient access to support which meet practical needs |  |
| **Health Status** | 6: Symptoms | Patient levels of pain or discomfort | Through direct interventions in a treatment or consultation room, through influencing any of the outcomes in the health empowerment domain, or by referring to other organisations who can provide further support in this. |
|  |  | Patient levels of anxiety, depression and stress |  |
|  |  | Other Signs and Symptoms |  |
|  |  | Side-effects of medication | Through listening to patients who wish to reduce medication due to side effects and developing a person-centred plan to achieve this where possible. |
|  | 7: Effect of symptoms on life | Patient ability to carry out normal daily life activities | By helping patients to reduce their symptoms (in the ways described above). Or, in cases where the symptoms cannot be reduced, by trying to understand the effect symptoms are having on the patients’ lives, and working with them to develop strategies to overcome difficulties. ‘Normal’ activities is a subjective construct. It may include paid employment, leisure activities, housework/DIY, shopping or anything involving acceptable levels of physical and mental function. |
|  |  | Patient ability to enjoy life |  |
|  |  | Impact of illness on other people in patients life |  |
| **Health Perceptions** | 8: Satisfaction with health | Patient perception of their health, in the context of their age and long-term conditions. | By helping patients to understand any conditions that they have, clearly communicating the scope and limitations of care and by ensuring that they always do not give up on persistent problems or symptoms. |
|  |  | Patient perception of damage to health resulting from poor care. | By ensuring they give the best care possible, including prompt diagnoses and referrals. By ensuring that they listen to patients and act on any warning signs. By always communicating clearly so that patients understand the risks and benefits of certain care options. |
|  | 9: Health Concerns | Patient concerns about serious illness | By listening to the patients and recognising when they may be harbouring concerns about serious illness or persisting symptoms. By taking these concerns seriously, examining the patient, ordering further tests if necessary and if not clearly explaining the rationale for any decisions and diagnoses. |
|  |  | Patient concerns about persisting symptoms |  |
|  |  | Patient unaddressed concerns | By taking time with patients to address all concerns, ensuring that, as far as is possible, patient do not leave primary care appointments with unaddressed concerns. By taking patients seriously and behaving in an approachable manner so patients feel comfortable raising all concerns. |
|  | 10: On track for the future | Patients belief that they are on the right path to addressing illness/problems | By ensuring that a clear plan of action is set for addressing problems. By listening to patients and ensuring that this path is one they feel willing and able to follow. |
|  |  | Patients belief that they are dealing with root causes of illness | By taking time with patients to establish the root causes of their problems, rather than offer ‘quick fixes’ of medication where this is not appropriate. Through clear explanations. |
|  |  | Patients confidence that clinicians do their best to detect serious illness | By always examining patients, taking every patient problem seriously and being thorough in any examination. |
